# p53 codon 72 polymorphism as a progression index for bladder cancer

**DOI:** 10.3892/or.2011.1610

**Published:** 2011-12-22

**Authors:** HUNG-YU LIN, CHUN-HSIUNG HUANG, TSAN-JUNG YU, WEN-JEN WU, MING-CHANG YANG, FOR-WEY LUNG

**Affiliations:** 1 Department of Urology, E-DA Hospital/I-SHOU University, Kaohsiung; 2 Graduate Institute of Medicine, College of Medicine, Kaohsiung Medical University, Kaohsiung; 3 Department of Urology, Kaohsiung Medical University, Kaohsiung; 4 Center for Genetic Research, Kaohsiung Armed Forces General Hospital, Kaohsiung; 5 Institute of Biological Science, National Sun Yat-Sen University, Kaohsiung; 6 Taipei City Psychiatric Center, Taipei City Hospital, Taipei; 7 Kaohsiung Armed Forces General Hospital, Kaohsiung; 8 National Defense Medical Center, Taipei, Taiwan, R.O.C

**Keywords:** genotype Pro/Pro, prognosis, cancer management index

## Abstract

The aim of this study was to calculate the positive predictive value (PPV) and negative predictive value (NPV) to determine whether p53 codon 72 can be used as a bladder cancer management index. Ninety-six patients diagnosed with bladed cancer and two control groups of 427 randomly sampled community participants and 142 non-cancerous individuals without a prior history of cancer were enrolled. After preliminary analysis, the convergent validity resulted in 96 patients from this study and 129 patients from our previous study. Results showed that these two groups were of the same population, and could be merged into one case group. Logistic regression showed that the Pro/Pro genotype was not statistically significantly associated with bladder cancer incidence using each sample set after adjustment by age and gender. Moreover, the Pro/Pro genotype was not associated with high-grade tumors (P=0.078), but was highly correlated to muscle-invasive tumors (P=0.002). Pro/Pro genotype carriers were estimated to have a 3.36-fold higher risk to develop invasive tumors compared to non-carriers. The NPV of the Pro/Pro genotype for invasive tumors was 88.00%, and the PPV was 31.91%. By Cox regression analysis, high-grade tumors were associated with recurrence (P=0.020, OR=1.83), whereas invasive tumors were associated with cancer-related death (P<0.001, OR=2.87). *p53* codon 72 polymorphism is associated with bladder cancer progression rather than incidence and prognosis. The Pro/Pro genotype in *p53* codon 72 polymorphism shows a high NPV for bladder cancer progression, thus, it can be used clinically as a progression index in bladder cancer management.

## Introduction

The TP53 gene is a well-known tumor suppressor gene, which serves as a transcriptional factor governing cell-cycle progression. TP53 promotes either growth arrest or apoptosis depending on the different stimuli and severity of DNA damage. The *p53* mutations have been found to be associated with oncogenesis in different cancer types; however, *p53* codon 72 polymorphism, a common nucleotide variation, is suggested to be involved in cancer vulnerability to cancers. We previously investigated the role of this polymorphism in colorectal cancer and reported that the genotype Pro/Pro was associated with disease progression. The genotype Pro/Pro carrier was estimated to have a 1.70-fold higher risk to develop more advanced tumors corresponding to each Duke stage (Dukes' A-D) (1). A previous report also suggested that the *p53* codon 72 polymorphism is involved in bladder invasiveness, staging and progression (2). Chen *et al* observed that Pro72 homozygosity was more frequent in bladder cancer patients, especially in those patients with invasive cancer (25 vs. 2.9%) (3). In our previous study, we proposed that the *p53* codon 72 polymorphism is involved in risk modulation of bladder cancer incidence and progression, but not prognosis, in Han Chinese (4). However, studies have shown discrepant findings about *p53* codon 72 polymorphism in bladder cancer, which make the association between this polymorphism and the bladder cancer risk inconclusive.

Thus, the purpose of this study was to validate whether the TP53 codon 72 variant alters tumor behavior during carcinogenesis of bladder cancer and modulates individual vulnerability in a new set of samples. Furthermore, if the TP53 codon 72 variant increases the risk for bladder cancer progression, the positive predictive value (PPV) and negative predictive value (NPV) were calculated to determine whether it can be used as a biomarker for bladder cancer progression index.

## Materials and methods

### Participants

This study was in compliance with the Helsinki Declaration and was approved by the Institutional Review Board at Kaohsiung Armed Forces General Hospital. A total of 96 patients, with an average age of 68.13 years (SD, 10.66), were included in this study. All patients were diagnosed as bladder cancer patients with corresponding histological examination data. The two groups of controls consisted of 427 randomly sampled individuals from the community, with an average age of 45.37 years (SD, 13.95), and of 142 non-cancerous individuals without a prior history of cancer, with an average age of 47.85 years (SD, 17.47), from the out-patient service of the Pain Clinic.

### Design and setting

Clinical and histopathological information were reviewed from the patients' medical charts. The stage of the tumors was categorized based on the presence of muscle invasion; thereby, the patients were classified into a non-muscle invasive (pTa, pT1, pTis) and muscle invasive (≥pT2d) group according to the 2004 WHO criteria (5). Moreover, the pathological grade was classified into a low grade (G1+G2) and a high grade (G3) according to the 2002 TNM classification. The age on the day of operation was used as the age of diagnosis. Cancer-related death was referred to death due to cancer or cancer-related complications, but trauma or other accidents were excluded.

### DNA extraction

Genomic DNA was extracted using the DNeasy Tissue kit (Qiagen, USA), and all the experimental procedures followed the manufacturer's protocol.

### Polymerase chain reaction/restriction fragment length polymorphism (PCR-RFLP)

Initially, intronic oligonucleotide primers spanning the SNP site were used to amplify the SNP-containing fragment, and the primer sequences were as follows: forward, 5′-CAA CGT TCT GGT AAG GAC AA-3′ and reverse, 5′-AAG CCT AAG GGT GAA GAG GAA-3′. PCR was performed in a final volume of 25 μl reaction buffer containing 50 mM KCl, 1.5 mM MgCl_2_, 10 mM Tris-HCl (pH 9.0), 2 μM EDTA, 0.1 mM DTT, 1% glycerol, 200 μM each of dATP, dCTP and dTTP, 50 μM dGTP, 150 μM 7-deaza-dGTP, 10 pmole of each primer, 1.5 U Taq polymerase, and 60 ng genomic DNA in 0.5 ml polypropylene microtubes, followed by a 35-cycle amplification (denaturation for 30 sec at 94˚C, annealing for 30 sec at 55˚C, and extension for 30 sec at 72˚C). Then, 10 μl amplified PCR products were subjected to *Bst*UI restriction enzyme digestion at 60˚C for 2 h. Afterwards, 8 μl of enzyme digested solution was mixed with 8 μl of 2X sample buffer (2X TBE, 0.1% bromophenol blue, 0.1% xylene cyanol, and 10% Ficoll 400), separated on a 2.5% non-denatured agarose gel (Amresco, USA) and visualized by ethidium bromide staining under a UV transilluminator. The product size of the Arg72 allele was 488 bp long, Pro72 allele-specific product was digested into 222 and 266 bp.

### DNA direct sequencing

PCR products were firstly purified using Qiaquick Purification Columns (Qiagen, USA), and cycle sequencing reaction was performed with a BigDye terminator cycle sequencing kit (Applied Biosystems, USA). The extended products were separated on an ABI Prism 3130 Genetic Analyzer (Applied Biosystems) after alcohol precipitation. The genotyping of the *p53* codon 72 polymorphism was duplicated, and two well-trained raters performed the genotype interpretation.

### Statistical analysis

The continuous variables such as age are indicated as mean ± SD. The genotyping data was treated as categorized variable. Data were analyzed using the SPSS 17.0 statistics software (SPSS Inc., USA). Independent sample t-test was used to test whether the intergroup difference in age between cases and controls was significant. The difference in gender distribution between the two groups was examined by the Fisher's exact test. The Pearson's χ^2^ test was applied to determine whether genotypic and allelic frequencies were significantly different between patients and controls. Multivariate logistic regression was implicated to determine the associated factor in relation to bladder cancer stage and grade. Finally, Cox's proportion hazard logistic regression was used to determine the associated factors of bladder cancer recurrence and cancer-related death.

## Results

After preliminary analysis, the results found that demographic variables of 96 patients were matched with 129 patients in our previous study (age, 65.54±11.84) (4). The convergent validity was established in these two groups (data not shown) and they could be enrolled into the same population, theoretically. In order to obtain a larger group of participants for calculating the clinical index, the two groups were merged into one group of participants.

The genotype and allele frequencies of p53 codon 72 polymorphism are shown in Table I. In sample set 1, no significant differences were found in the genotype and allele frequencies between cases and controls (genotype, P=0.100; allele, P=0.613). In contrast, both genotypic and allelic frequencies of *p53* codon 72 polymorphism were significantly different between the bladder cancer cohort 2 and the non-cancerous controls (genotype, P=0.017; allele, P=0.014), in which the genotype Pro/Pro and Pro allele was more frequent in patients than in controls (genotype Pro/Pro, 24.47 vs. 18.31%; C allele, 53.72 vs. 42.25%). All genotype distributions of each sample set were consistent to a Hardy-Weinberg equilibrium (HWE) (P>0.05). No significant differences were found in either genotype distribution or allele frequency between the merged patient population and the control group (genotype, P=0.292; allele, P=0.208). However, in both sample set 2 and in the merged sample set, the age and gender distribution were all evidently different between cases and controls (sample set 2, age, t=9.97, P<0.001; gender, P<0.001; merged sample set, t=−17.93, P<0.001; gender, P<0.001). Additionally, the frequencies of Pro homozygosity was higher in the patients than in the controls among all three sample sets (sample set 1, 20.93 vs. 17.33%; sample set 2, 24.47 vs. 18.31%, merged samples, 22.42 vs. 17.57%); thereby, the genotype Pro/Pro was viewed as risk allele, multivariate logistic regression was preceded to investigate the possible effect of *p53* codon 72 polymorphism in bladder cancer incidence with age and gender adjusted.

Using logistic regression, the role of the genotype Pro/Pro involved in bladder cancer incidence was tested individually in sample sets 1 and 2 with age and gender as adjusting factors, as shown in Table II. Genotype Pro/Pro had no effect on bladder cancer incidence in neither sample set (sample set 1, P=0.867, sample set 2, P=0.926). Since both sample sets showed similar demographics and same results in logistic regression analysis, they were merged to form the merged sample set. Using the merged sample set, with the age and gender adjusted, genotype Pro/Pro was not statistically-significantly associated with bladder cancer (P=0.954).

To investigate the correlation between *p53* codon 72 polymorphism and bladder cancer progression, the genotype Pro/Pro was analyzed independently for its association with staging and grading using age and gender as covariates, as shown in Table II. There was no significant correlation between diagnostic age, gender, tumor stage, and grade (P>0.05). The genotype Pro/Pro was significantly associated with staging (P=0.002), but only in relation to grading with a borderline significance (P=0.078). The PPV of the genotype Pro/Pro to bladder cancer staging was 31.91% and the NPV was 88.00%. Thus, patients homozygous for the Pro allele were more likely to have a muscle-invasive tumor (OR=3.36) rather than a high-grade tumor (Table II). In addition, the non-genotype Pro/Pro carriers were frequent to have low-stage tumors than genotype Pro/Pro carriers.

Cox's regression analysis showed that the patients with high-grade tumors (tumor grade, P=0.020, OR=1.83, 95% CI=1.10–3.03) were more likely to have recurrent tumor compared to those patients with low-grade tumor, as shown in Table III. Moreover, patients with muscle-invasive tumors (tumor stage, P<0.001, OR=2.87, 95% CI=1.69–4.86) were more likely to have a shorter survival time compared to those patients with low-stage tumors. However, the extent of the *p53* codon 72 polymorphism may not predict either recurrence or patient survival (P>0.05) (data not shown).

## Discussion

In addition to the sample set used in the previous study (4), a new set of sample was recruited, and the results partially replicated our previous findings of genotype Pro/Pro of *p53* codon 72 polymorphism associated with bladder cancer staging (4), but with age and gender controlled, the results did not support its role in risk modulation of bladder cancer incidence. In contrast, the *p53* codon 72 polymorphism does not serve as a biomarker to predict bladder cancer prognosis regardless of the issues of recurrent tumor or cancer-related death. The genotype Pro/Pro carrier was estimated to have a 3.36-fold increased risk to develop muscle-invasive bladder cancer compared to the non-carriers. With an NPV of 88%, genotype Pro/Pro may be used as a progression index in bladder cancer.

Regarding the biological functions of TP53 and cancer, it is believed that loss of wild-type p53 expression will lead to a disability of cell-cycle control, as well as failure to induce efficient apoptosis. Most of the cancer types were detected with various *p53* mutations, and these mutations directly associated with abnormalities of wild-type p53. Of those *p53* mutations and/or common variations, codon 72 polymorphism is documented to confer risk modulation of individual vulnerability to bladder cancer (6) and/or advanced disease (7). In fact, a previous study has suggested that those two codon 72 variants exhibit different ability to trigger apoptosis at the cellular level (8), which concluded that the Arg72 variant induced apoptosis more effectively than the Pro72 variant (9). In contrast, the Pro72 variant has also been reported to cause a G1-arrest-like phenotype (10). Unlike the reported frequencies of codon 72 polymorphism of the patient cohort in our previous study (4), this study used the genotyping data of this cohort without adjustment by the *p53* loss of heterozygosity (LOH) status in the performed analyses. In all sample sets (sample sets 1, 2 and the merged sample set), the genotype Pro/Pro was more frequently observed in the cases than in the controls. Especially the second sample set demonstrated a significant intergroup difference in both genotypic and allelic frequencies. Although the genotype distribution of both groups was consistent to HWE; nevertheless, except the gender distribution of sample set 1, the distributions of age and gender between case and control groups are quite different; therefore, it was adjusted in the logistic regression analysis to investigate the possible role of *p53* codon 72 polymorphism in bladder cancer incidence. Based on our data, we suggested that *p53* codon 72 polymorphism is unlikely to be associated with bladder cancer incidence, but correlated to progression. In most types of cancer, the Pro allele and/or the genotype Pro/Pro detection frequencies are higher than in the control groups (1,11). Previous studies have found that the bladder cancer, unlike other cancers, the Arg72 allele was more frequent and was associated with bladder cancer incidence (2,12). Nevertheless, other studies have reported inconsistent results (7,11). The reason for the discrepancies in the findings of whether *p53* codon 72 polymorphism confers vulnerability to bladder cancer may include the ethnic heterogeneity, the sample types (blood vs. tumor tissue), the presence of HPV-DNA, the sample selection bias, and the cancer pathological differences. The rationale is that tumors harboring the Arg72 allele may be the consequence of tumor clonal expansion under selective pressure during bladder carcinogenesis (12,13). The tumors with Arg72 allele may correlate to HPV and make them more vulnerable to viral infection (14. Viral E6 protein was found to interact with Arg72 variant and resulted in lowering down p73-mediated apoptotic activity (15). On the other hand, the genotype Pro/Pro was more highly associated with muscle invasive stage rather than high-grade tumors. In this study, the genotype only demonstrated borderline significant association with grade. However, we hypothesize that this result might be due to our small sample size of bladder cancer participants, resulting in insufficient power to identify the association between stage and the genotype Pro/Pro. Accordingly, those patients harboring genotype Pro/Pro were estimated to have a 3.36-fold elevated risk to develop muscle-invasive tumors compared to the non-genotype Pro/Pro genotype carrier (genotype Arg/Arg and Arg/Pro). In the logistic regression, carrying genotype Pro/Pro predicts 31.91% of invasive bladder cancers in a parsimonious model. Overall, to predict the invasive bladder tumor, PPV of genotype Pro/Pro was 31.91% and NPV 88.00%, which was better than that of the genotype Arg/Arg and Arg/Pro (data not shown). Therefore, detecting *p53* codon 72 polymorphism using patients' blood may provide a modest predictive value for bladder cancer progression. Our findings are consistent with a previous study, in which the genotype Pro/Pro was shown to be more frequent in invasive bladder tumors (≥pT2) compared to the superficial tumor in Japanese population (19.6 vs. 14.8%). Moreover, the genotype Pro/Pro carriers were shown to have a significantly poorer survival and higher risk of disease-specific death than the genotype Arg/Pro and Arg/Arg carrier after radial cystectomy (16). Taken together, these results indicate that the genotype Pro/Pro was more likely to be associated with invasive bladder tumors than other genotypes.

Logistic and Cox's regression analysis found associations between genotype Pro/Pro and stage, grade and recurrence, stage and cancer-related death. However, the genotype Pro/Pro was only intermediately correlated to bladder cancer staging (r=0.244) based on the coefficients of squared multiple correlation in our model using structure equation modeling (data not shown). The results did not support that the genotype Pro/Pro can be a biomarker for predicting bladder cancer prognosis. However, since previous studies have mentioned that cancer prognosis was determined by multiple factors such as psychosocial, environmental and emotional problems (17,18), the hypothesis is reasonable. Therefore, a psychosocial survey may be needed when studying the issue of cancer prognosis. Despite the fact that the *p53* codon 72 polymorphism showed no value in predicating bladder cancer prognosis, the good NPV of genotype Pro/Pro to bladder cancer staging may be useful in bladder cancer management, especially for the purpose of risk assessment.

Using *p53* codon 72 polymorphism as a progression index in bladder cancer resulted in a PPV of 31.91% and an NPV of 88%. Although the PPV is lower, it is more sensitive to prevalence than NPV. Thus, in primary care medical settings where the prevalence is low, NPV is more clinically relevant (19). With an NPV of 88%, *p53* codon 72 polymorphism can be used as a progression index for bladder cancer, meaning that those genotype Pro/Pro carriers of *p53* codon 72 polymorphism are not likely to progress to an invasive cancer.

However, this study has some limitations. First, it is a retrospective study rather than a prospective one. Thus, the clinical information such as smoking status, co-morbidities, and medical records may not be complete. Second, known risk factors of bladder cancer such as smoking, mutagen exposure, water drinking, familial cancer history and social-economical factors were not fully controlled when studying the association between *p53* codon 72 polymorphism, bladder cancer incidence and progression; and thus, these factors may be latent factors overthrowing current findings. Third, benign tumors were not included. Only the single polymorphism of *p53* was investigated which may not fully illustrate its role involved in cancer incidence, progression and prognosis (20,21). A further study including other *p53* SNPs or neighboring loci that have a high linkage disequilibrium (LD) with *p53* will be worthy of investigation. Fourth, for survival analysis, only the survival patients would revisit our clinics and possibly be recruited and followed-up; therefore, a length-time bias (selection bias) probably occurred and resulted in a type I error when investigating the association between *p53* codon 72 polymorphism and bladder cancer prognosis. The patients would not be followed-up resulting in a lack of information about recurrent tumors and/or the recurrence of cancer-related death if they did not revisit due to private factors or due to unknown causes. Finally, the types of chemotherapeutic drug was not controlled, therefore, the response, mutual influence and common functions of different types of anticancer drugs were not taken into consideration. Simultaneously, patient information on dietary intake, smoking status, financial circumstances, individual professional status, and other co-morbidities were not controlled; these may be latent factors that affect the success of bladder cancer therapy and/or cancer recurrence, progression and anti-cancer drug response.

*p53* codon 72 polymorphism is associated with bladder cancer progression. The genotype Pro/Pro carrier increased 3.36-fold the risk to develop invasive tumors by compared to non-carrier. The patients with high-grade primary tumors have a 1.83-fold higher risk to develop recurrent tumors than patients with low-grade primary tumors. Moreover, patients with muscle-invasive tumors have a 2.87-fold higher risk to succumb to the disease than low-stage tumor victims. With a high NPV, the genotype Pro/Pro allele can be used clinically as a progression index in bladder cancer. The results of our new sample presented similar results to the previous study (4), showing good external validity and a generalizable ability to the Han Chinese population.

## Figures and Tables

**Table I:** The frequencies of *p53* codon 72 polymorphism between bladder cancer subjects and controls.

Sample set 1	Bladder cancer cohort 1 (n=129)	Community controls (n=427)	t-value	χ^2^	P-value
Age, mean ± SD	65.54±11.84	45.37±13.95	13.81		<0.001
Males, n (%)	57/108 (52.78)^a^	188/427 (44.03)			0.106
Codon 72 genotype, n (%)					
CC	27 (20.93)	74 (17.33)			
CG	55 (42.64)	228 (53.40)			
GG	47 (36.43)	125 (29.27)		4.60	0.100
HWE-χ^2^	P=0.151	P=0.085			
Allele, n (%)					
C	109 (42.25)	376 (44.03)			
G	149 (57.75)	478 (55.97)		0.26	0.613
Sample set 2	Bladder cancer cohort 2 (n=96)	Non-cancerous controls (n=142)	t-value	χ^2^	P-value
	
Age, mean ± SD	68.13±10.66	47.85±17.47	9.97		<0.001
Male, n (%)	68/90 (75.56)^b^	52/140 (37.14)^b^			<0.001
Codon 72 genotype, n (%)^c^					
CC	23 (24.47)	26 (18.31)			
CG	55 (58.51)	68 (47.89)		8.13	0.017
GG	16 (17.02)	48 (33.80)			
HWE-χ^2^	P=0.086	P=0.824			
Allele, n (%)					
C	101 (53.72)	120 (42.25)			
G	87 (46.28)	164 (57.75)		5.98	0.014
Merged sample set	Combined bladder cancer (n=225)	Combined normal population (n=569)	t-value	χ^2^	P-value
	
Age, mean ± SD	66.73±11.36	45.98±14.91	−17.93		<0.001
Males, n (%)	125/201 (62.19)^d^	240/567 (42.33)^d^			<0.001
Codon 72 genotype^e^					
CC	50 (22.42)	100 (17.57)			
CG	110 (49.33)	296 (52.02)		2.46	0.292
GG	63 (28.25)	173 (30.41)			
HWE-χ^2^	P=0.880	P=0.168			
Allele					
C	210 (47.09)	496 (43.59)			
G	236 (52.91)	642 (56.41)		1.59	0.208

a Twenty-one patients without gender information;

b 6 bladder cancer patients and 2 non-cancerous controls without gender information;

c 2 bladder cancer patients without genotyping data;

d 24 patients and 2 controls without gender information;

e 2 bladder cancer patients without genotyping data.

HWE, Hardy-Weinberg equilibrium.

**Table II:** Multivariate logistic regression to determine the associated factors of bladder cancer incidence, staging and grading.

Dependent variable: bladder cancer incidence	Sample set 1 (n=556)							
	
	Mean or count (%)	B	SE	P-value	Exp(B)	95% CI		
	
Males	245/535 (45.79)^a^	−0.216	0.259	0.405	0.81	0.49–1.34		
Age	49.45±15.78	0.101	0.010	<0.001	1.11	1.08–1.13		
Genotype Pro/Pro	101/556 (18.17)	−0.055	0.325	0.867				
Constant		−6.638	0.741	<0.001	0.001			
Dependent variable: bladder cancer incidence	Sample set 2 (n=238)							
	
	Mean or count (%)	B	SE	P-value	Exp(B)	95% CI		
	
Males	120/230 (52.17)^b^	−1.640	0.362	<0.001	0.19	0.10–0.39		
Age	55.91±18.18	0.089	0.013	<0.001	1.09	1.06–1.12		
Genotype Pro/Pro	49/236 (20.76)^c^	0.039	0.425	0.926				
Constant		−3.340	0.907	<0.001	0.04			
Dependent variable: bladder cancer incidence	Merged sample (n=792)							
	
	Mean or count (%)	B	SE	P-value	Exp(B)	95% CI		
	
Males	365/768 (47.53)^d^	−0.693	0.204	<0.001	0.50	0.34–0.75		
Age	51.40±16.76	0.098	0.008	<0.001	1.10	1.09–1.12		
Genotype Pro/Pro	150/792 (18.94)	0.014	0.250	0.954				
Constant		−5.587	0.560	<0.001	0.004			
Dependent variable: stage	Bladder cancer subjects (n=194)							
	
	Mean or count (%)	B	SE	P-value	Exp(B)	95% CI	PPV	NPV
	
Males	118/194 (60.82)	0.175	0.306	0.567				
Age	66.95±11.38	−0.013	0.013	0.325				
Genotype Pro/Pro	42/194 (21.65)	1.211	0.386	0.002	3.36	1.58–7.15	31.91	88.00
Constant		0.308	0.998	0.758				
Dependent variable: grade								
Gender		0.002	0.315	0.994				
Age		0.007	0.013	0.586				
Genotype Pro/Pro		0.715	0.406	0.078				
Constant		−0.119	1.013	0.906				

a Twenty-one participants without gender information;

b 8 participants without gender information;

c 2 patients without genotyping data;

d 24 patients have no gender information;

PPV, positive predicting value; NPV, negative predicting value.

**Table III:** Cox's regression to determine the associated factors of bladder cancer prognosis.

Dependent variable: recurrence	Bladder cancer patients (n=179)^a^				
	
	B	SE	P-value	Exp(B)	95% CI
	
Grade	0.602	0.258	**0.020**	**1.83**	**1.10–3.03**
Dependent variable: cancer-related death	Bladder cancer patients (n=183)^b^				
	
	B	SE	P-value	Exp(B)	95% CI
	
Stage	1.053	0.269	**<0.001**	**2.87**	**1.69–4.86**

a Fifteen patients without recurrence data;

b 11 patients without follow-up information.
